# Development and Evaluation of a Quality Assessment Tool for Laparoscopic Sleeve Gastrectomy Videos: A Review and Comparison of Academic and Online Video Resources

**DOI:** 10.1007/s11695-024-07199-0

**Published:** 2024-04-06

**Authors:** Laith Alghazawi, Michael G. Fadel, Jun Yu Chen, Bibek Das, Henry Robb, Maria Rita Rodriguez-Luna, Naim Fakih-Gomez, Silvana Perretta, Hutan Ashrafian, Matyas Fehervari

**Affiliations:** 1 Department of Surgery and Cancer, Imperial College London, London, UK; 2 Department of Bariatric and Metabolic Surgery, Chelsea and Westminster Hospital, London, UK; 3 Research Institute Against Digestive Cancer (IRCAD), Strasbourg, France; 4 ICube Laboratory, Photonics Instrumentation for Health, Strasbourg, France; 5 Department of Digestive and Endocrine Surgery, University of Strasbourg, Strasbourg, France; 6 IHU-Strasbourg, Institute of Image-Guided Surgery, Strasbourg, France; 7 Gastrointestinal Surgery, Maidstone and Tunbridge Wells NHS Trust, Tunbridge Wells, UK

**Keywords:** Laparoscopic sleeve gastrectomy, Educational video, Video reporting guidelines, Video quality assessment

## Abstract

**Background:**

Video recording of surgical procedures is increasing in popularity. They are presented in various platforms, many of which are not peer-reviewed. Laparoscopic sleeve gastrectomy (LSG) videos are widely available; however, there is limited evidence supporting the use of reporting guidelines when uploading LSG videos to create a valuable educational video.

We aimed to determine the variations and establish the quality of published LSG videos, in both peer-reviewed literature and on YouTube, using a newly designed checklist to improve the quality and enhance the transparency of video reporting.

**Methods:**

A quality assessment tool was designed by using existing research and society guidelines, such as the Bariatric Metabolic Surgery Standardization (BMSS). A systematic review using PRISMA guidelines was performed on MEDLINE and EMBASE databases to identify video case reports (academic videos) and a similar search was performed on the commercial YouTube platform (commercial videos) simultaneously. All videos displaying LSG were reviewed and scored using the quality assessment tool. Academic and commercial videos were subsequently compared and an evidence-based checklist was created.

**Results:**

A total of 93 LSG recordings including 26 academic and 67 commercial videos were reviewed. Mean score of the checklist was 5/11 and 4/11 for videos published in articles and YouTube, respectively. Academic videos had higher rates of describing instruments used, such as orogastric tube (*P* < 0.001) and stapler information (*P* = 0.04). Fifty-four percent of academic videos described short-term patient outcomes, while not reported in commercial videos (*P* < 0.001). Sleeve resection status was not universally reported.

**Conclusions:**

Videos published in the academic literature are describing steps in greater detail with more emphasis on specific technical elements and patient outcomes and thus have a higher educational value. A new quality assessment tool has been proposed for video reporting guidelines to improve the reliability and value of published video research.

**Graphical abstract:**

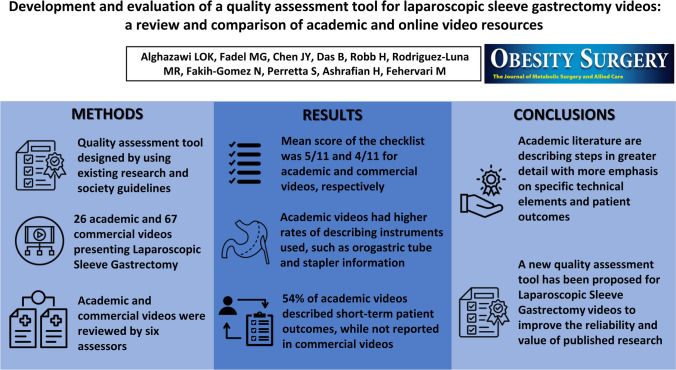

**Supplementary Information:**

The online version contains supplementary material available at 10.1007/s11695-024-07199-0.

## Introduction

Video recordings of surgical procedures have been increasingly used for training, demonstration of new techniques, presentation of unique cases or determination of outcomes, as well as for legal reasons [1, 2]. Laparoscopic sleeve gastrectomy (LSG) is one of the most common procedures in bariatric surgery [3, 4] and many academic and commercial videos have been published. Video analysis of LSG technique identified associations between technical variations and postoperative complications, including staple line leak and haemorrhage rates [5]. These findings highlight the importance of operative videos in measuring surgical quality. Recordings of operations are also utilised for educational purposes and play an important role in simulation training [1, 6].

Video recordings have the advantage of demonstrating operative skills remotely, allowing surgeons in training to use videos to prepare for operations and quickly learn skills and procedures [2, 7]. Additionally, it permits retrospective review and monitoring of progression. Consequently, there is growing evidence that videos enhance surgical skills and techniques [1, 8] and improve surgical training [9]. From a clinical perspective, videos can link postoperative complications to intraoperative techniques and identify errors [5]. These recordings are presented in various methods, including academic publications (e.g., video case reports), on commercial platforms (e.g., YouTube) and educational resources [2, 10]. However, these platforms present videos in different form such as full length, highlights, narrated or with subtitles, and without audio. Heterogenous reporting of videos can reduce the educational value of them, impair the communication of the intended learning or clinical outcome and limit the possibility of auditing them.

The steps of a gold standard of LSG procedure have been established by expert consensus in the Bariatric Metabolic Surgery Standardization (BMSS) [11] to improve consistency in surgery, data collection and outcome reporting. However, the utilisation of these guidelines in practice and the demonstration of best practice in academic and commercial videos is unknown. Our aim was to determine the quality of both academic and commercial videos by using a checklist based on BMSS guidelines and describe the degree of heterogeneity in both peer-reviewed publications and LSG videos on YouTube.

## Methods

### Search Strategy

A literature search of MEDLINE (via PubMed) and EMBASE (via OVID) was performed in January 2023 using Preferred Reporting Items for Systematic Reviews and Meta-Analyses (PRISMA) guidelines [12]. Studies were identified by using the search terms: ((Laparoscopic Sleeve Gastrectomy OR Sleeve Gastrectomy OR Bariatric Surgery OR Metabolic Surgery OR Bariatric Surgical Procedures OR Weight Loss Surgery OR Stomach Stapling OR Stapling, Stomach OR Gastrectomy) AND “Video-Audio Media” [pt]). A further search was made on the YouTube website for LSG videos by using the search term: (Laparoscopic Sleeve Gastrectomy). Inclusion and exclusion criteria were specified for both peer-reviewed articles and YouTube videos, and are described as the following:

Inclusion criteria:

I) Articles with attached televisual media.
II) Videos that show the beginning and end of the LSG procedure (not part of procedure).
III) YouTube videos uploaded in 2021 and 2022.

Exclusion criteria:

I) LSG was not the primary surgical procedure being recorded.
II) Video in the peer-reviewed articles that were unavailable or not accessible.
III) Animated or duplicate videos.

### Checklist Development

The BMSS World Consensus Meeting defined standard anatomic measurements for each bariatric procedure through expert consensus in 2018 [11]. They aimed to propose a high-quality standardisation of dimensions and volumes for the procedure’s key anatomic variations, including LSG. The core elements identified during the BMSS consensus for LSG procedure included distance of sleeve transection from the gastro-oesophageal junction (GOJ) and pylorus, the length of the sleeve, the use of an appropriately sized orogastric tube and the volume of the sleeve. BMSS guidance deemed these critical steps necessary to ensure safety and optimal outcomes for patients undergoing LSG. Furthermore, a recent study by Chhabra et al. [5] prospectively reviewed LSG videos in a large cohort of videos, showing significance between postoperative patient outcomes and video evaluation of surgical techniques. The study provided a breakdown of the aspects of LSG operations, the clinical significance of these important aspects and their corresponding peer-rated components. The majority of the identified components were similar to the BMSS guidance, however, included other parameters such as leak test demonstration and dissection of proximal stomach (complete mobilisation of fundus, visualisation of left crus and complete division of short gastric vessels).

There is consensus on key components needed in laparoscopic recorded procedures for educational purposes. LAParoscopic surgery Video Educational GuidelineS (LAP-VEGaS) practice guidelines [13] reported the importance of illustrating the case presentation first, demonstrating the surgical procedure, and describing patient outcomes within each video. Following this, the LAP-VEGaS was used to evaluate LSG videos on YouTube by Chapman et al. [14]. However, they did not specifically examine the LSG procedure step-by-step.

**Fig. 1:**
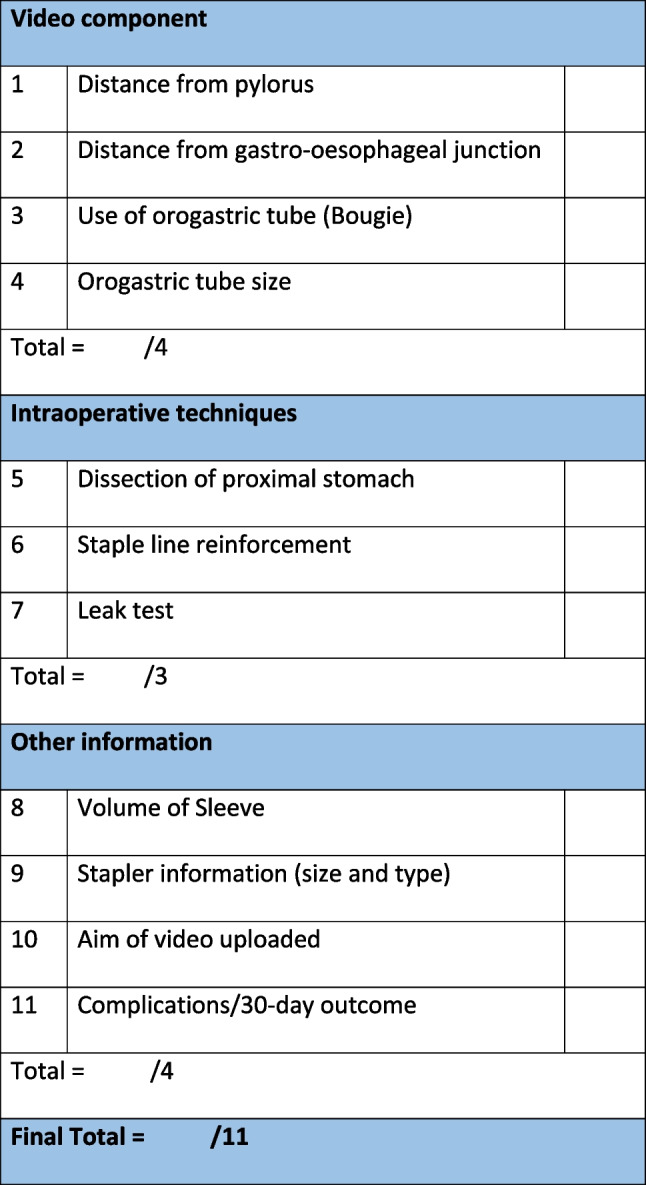
Reporting checklist for laparoscopic sleeve gastrectomy video quality assessment

The checklist was developed using the gold standard steps of LSG described in BMSS and supplementing it with data from the literature (Chhabra et al. study and LAP-VEGaS). We identified three important elements of videos describing LSG as displayed in Fig. 1. The first element, “Video component”, are agreed key procedurals skills in LSG, shared between Chhabra et al. and the BMSS consensus [8, 9]. The “intraoperative technique” section of the checklist was developed from the literature connecting postoperative patient outcomes and intraoperative surgical techniques performed [5]. The last component titled “Other information” are aspects not related to the recording itself, but rather are of educational and clinical significance, supported by the LAP-VEGaS consensus [13]. A total of 11 fields were identified as part of the video reporting checklist, corresponding to one point each. Video length and audio status (narrated, music, no audio) were also collected for analysis. They contained each key surgical step within all LSG procedures, as well as other domains within the video that determined the end quality of the published surgical videos. Median (range) values are presented in the text, unless stated otherwise.

### Video Data Extraction, Marking, and Statistical Analysis

Each video included in the analysis was subjected to evaluation by the reporting checklist. Videos from included articles were reviewed independently by six reviewers (LA, MGF, JYC, HR, NFG and MF) assessed against each domain of the checklist. Of those six reviewers, two were junior surgical trainee (surgical training year 1–3), two were at a senior surgical trainee (surgical training year 4–8,) and two were at consultant level.

Statistical analysis was performed with SPSS for Mac OSX 21.0.0 (SPSS Inc., Chicago, IL) statistical software. As many of the variables had non-Gaussian distributions, we used non-parametric tests for the analysis. Statistical analyses were performed using a two-tailed test and *P* < 0.05 was considered statistically significant. Inter-rater reliability of the checklist marking was performed using Cohen’s kappa coefficient and the Fisher exact test was used to assess the difference in narrations between academic and commercial videos.

## Results

**Fig. 2:**
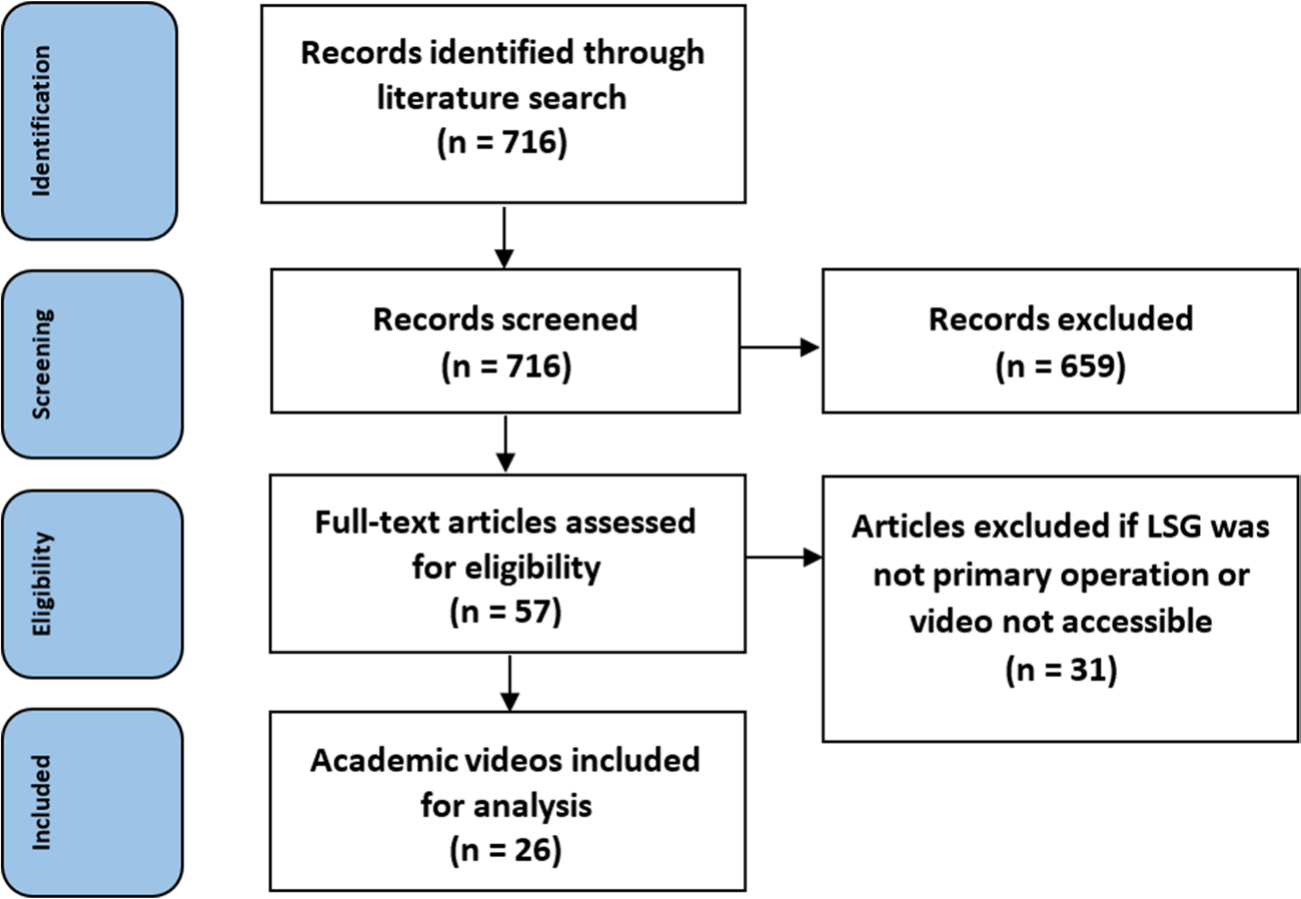
The flowchart shows the peer-reviewed video-based literature search and study selection process according to the PRISMA guidelines. *LSG* laparoscopic sleeve gastrectomy

**Fig. 3:**
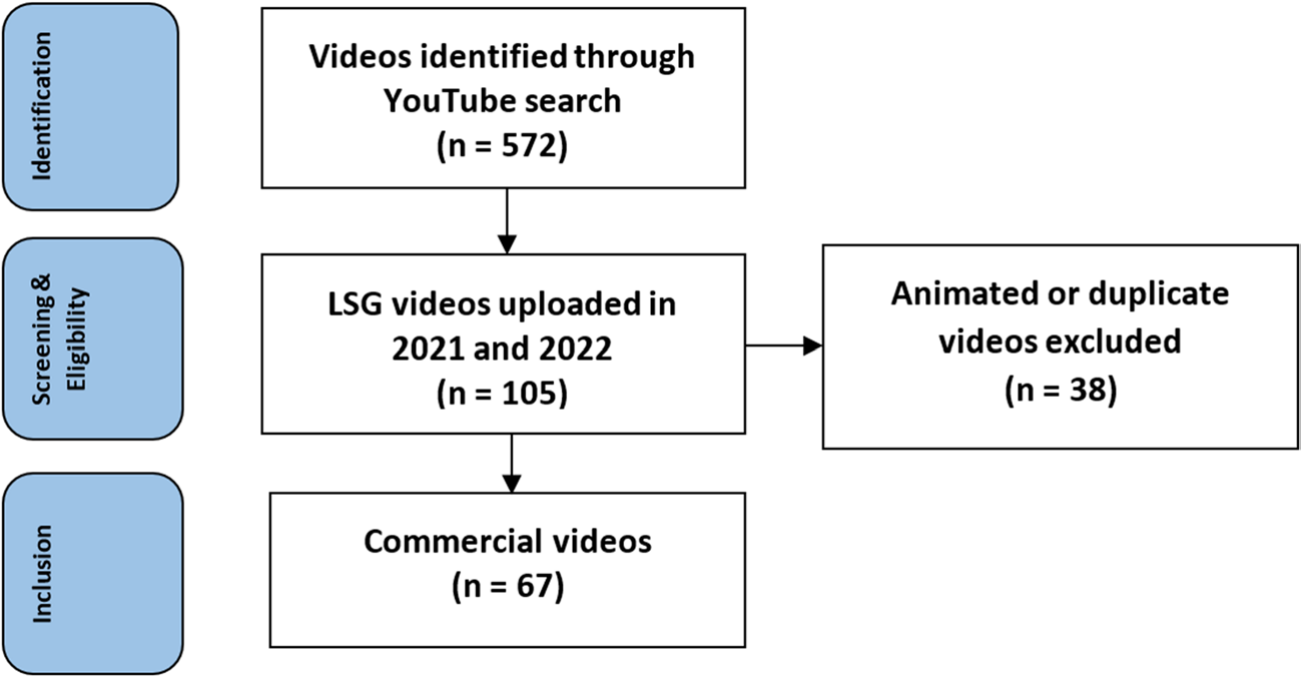
The flowchart demonstrates the selection process for the YouTube videos related to Laparoscopic Sleeve Gastrectomy. *LSG* laparoscopic sleeve gastrectomy

A total of 716 articles were identified. The flowchart of the search is presented in Figs. 2 and 3. Overall, twenty-six academic videos and sixty-seven commercial videos were included in the analysis.

The median time of the LSG videos was 13 min and 43 s. Specifically, videos uploaded in peer-reviewed literature had a median time of 7 (5–12) minutes whilst LSG videos uploaded to YouTube had a median time of 17 (2–120) minutes.

The total median score for academic videos was 5 (1–10) and 4 (1–10) out of a total of 11 for academic and commercial videos, respectively. None of the videos analysed, either peer-reviewed or on YouTube, described the volume of the resected sleeve specimen or gained a full score of 11. Table 1 shows the percentages and scores for each component of the developed checklist, comparing academic and commercial videos.

**Table 1:** Checklist and analysis of laparoscopic sleeve gastrectomy videos from academic and commercial video platforms. GOJ, gastro-oesophageal junction. * = statistically significant

	Total videos (n = 93)		Academic videos (*n* = 26)			Commercial videos (*n* = 67)		
Parameter	Yes (%)	No (%)	Yes (%)	No (%)	Yes (%)		No (%)	*P*-Value
Distance from pylorus	38 (41)	55 (59)	11 (42)	15 (58)		27 (40)	40 (60)	1
Distance from GOJ	68 (73)	25 (27)	20 (77)	6 (23)		48 (72)	19 (28)	0.79
Use of orogastric tube	84 (90)	9 (10)	24 (92)	2 (8)		60 (90)	7 (10)	1
Orogastric tube size (when used)	29 (35)	55 (65)	22 (92)	2 (8)		7 (12)	53 (88)	< 0.001*
Dissection of proximal stomach	74 (80)	19 (20)	16 (62)	10 (38)		58 (86)	9 (14)	0.011*
Staple line reinforcement	48 (51)	45 (49)	16 (62)	10 (38)		31 (46)	36 (54)	0.25
Leak test	24 (26)	69 (74)	8 (31)	18 (69)		16 (24)	51 (76)	0.59
Volume of sleeve	0 (0)	93 (100)	0 (0)	26 (100)		0 (0)	67 (100)	1
Stapler information (size and type)	48 (52)	45 (48)	18 (69)	8 (31)		30 (45)	37 (55)	0.04*
Aim of video uploaded	93 (100)	0 (0)	26 (100)	0 (0)		76 (100)	0 (0)	1
Complications / 30-day outcome	9 (10)	84 (90)	14 (54)	12 (46)		0 (0)	67 (100)	< 0.001*

All videos describe their aim or reason for uploading the video, which was primarily demonstrative or educational. None of the YouTube videos described short-term outcomes, whereas half of the peer-reviewed articles did. High percentage of agreement and good inter-rater reliability were demonstrated between the six assessors from various level of expertise. Regardless of the level of expertise there was an agreement in nearly all elements of the checklist, except for distance from pylorus, as demonstrated on Table 2. The raw total score for each assessor is provided in Supplementary File 1.

**Table 2:** Inter-rater reliability, both overall and among groups of surgeons with varying levels of expertise

	Overall			Junior surgical trainees			Senior surgical trainees			Consultants		
Score parameter	Percentage agreement	Feiss's kappa	*P* value	Percentage agreement	Cohen's kappa	*P* value	Percentage agreement	Cohen's kappa	*P* value	Percentage agreement	Cohen's kappa	*P* value
Distance from pylorus	33.3	0.403	< 0.001	57.1	0.219	0.0116	83.3	0.67	< 0.001	94	0.876	< 0.001
Distance from GOJ	48.8	0.516	< 0.001	95.2	0.88	< 0.001	86.9	0.738	< 0.001	91.7	0.832	< 0.001
Use of orogastric tube	90.5	0.808	< 0.001	100	1	< 0.001	97.6	0.909	< 0.001	100	1	< 0.001
Orogastric tube size	76.2	0.729	< 0.001	97.6	0.943	< 0.001	95.2	0.881	< 0.001	91.7	0.775	< 0.001
Dissection of stomach	79.8	0.537	< 0.001	96.4	0.881	< 0.001	92.9	0.629	< 0.001	98.8	0.917	< 0.001
Staple line reinforcement	71.4	0.75	< 0.001	94	0.881	< 0.001	90.5	0.809	< 0.001	100	1	< 0.001
Leak test	79.8	0.719	< 0.001	96.4	0.903	< 0.001	98.8	0.968	< 0.001	94	0.833	< 0.001
Volume of sleeve	85.7	0.314	< 0.001	100	NaN	NaN	96.4	0.65	< 0.001	92.9	0.466	< 0.001
Stapler info	64.3	0.652	< 0.001	96.4	0.928	< 0.001	95.2	0.902	< 0.001	97.6	0.952	< 0.001
Aim of video	85.7	0.0793	0.0049	100	NaN	NaN	90.5	-0.0213	< 0.001	96.4	0.556	< 0.001
Complications	92.9	0.873	< 0.001	100	1	< 0.001	98.8	0.96	< 0.001	100	1	< 0.001

**Fig. 4:**
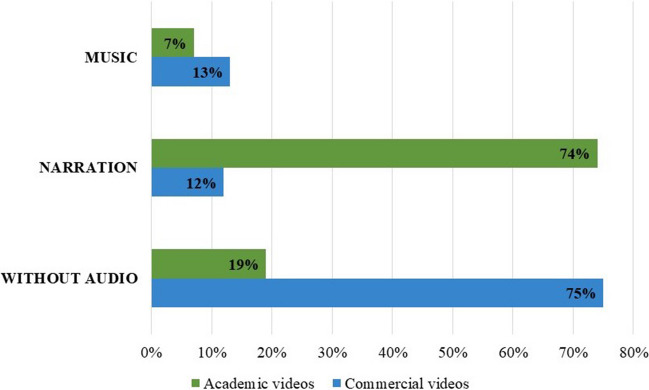
Analysis of the use of sound in laparoscopic sleeve gastrectomy videos from academic and commercial video platforms

There were notable differences in the type and frequency of audio elements. Narration was more prevalent in academic videos, whereas commercially available videos often lacked audio or used music instead of formal narration to describe the operation (Fig. 4). Statistically significant differences were observed in the use of narration (*P* < 0.001) and the absence of audio (*P* < 0.001) between the two groups. However, no statistically significant differences were identified in the use of music between academic and commercial videos (*P* = 0.72), as demonstrated in Table 3.

**Table 3:** Comparison of music, narration, and audio in laparoscopic sleeve gastrectomy videos from academic and commercial video platforms. * = statistically significant

	Academic videos (%)	Commercial videos (%)	*P* value
Music	2 (7%)	9 (13%)	*P* < 0.72
Narration	19 (74%)	8 (12%)	*P* < 0.001*
Without Audio	5 (19)	50 (75%)	*P* < 0.001*

## Discussion

The analysis demonstrated inconsistent reporting in the videos uploaded in both peer-reviewed literature and the YouTube platform. Videos published in the academic literature had a greater tendency to describe technical information in detail. They provided information regarding the surgical instruments used and patient outcomes. There was no audio or background music found in more than 85% of YouTube videos, compared to 26% of academic videos. There is potentially higher educational value in narration, for example, through the expert description of surgical instrumentation. This is supported by the LAP-VEGaS consensus statement [10]. The findings of this study therefore suggest that videos clips in the academic literature have more educational value than commercial platforms.

Further analysis revealed that all videos failed to mention the resected volume of the sleeve. The exact importance of this has not yet been established. There is some evidence in literature to support the relationship between resected sleeve volume and weight loss [15–17], although other authors have disputed this and described the resected volume to be related to the initial body mass index, rather than as a predictor of weight loss [18, 19].

This qualitative review has led to the development of an evidence-based checklist for LSG video reporting using BMSS guidance. Although there are no established video reporting guidelines yet, checklists are routinely used in the literature to assess observational studies, systematic reviews and meta-analyses. For example, the STROBE statement comprises of a checklist of items that should be included in articles reporting observational research [20] and the MOOSE checklist contains specifications for reporting meta-analyses of observational studies in epidemiology [21]. To our knowledge, this is the first reporting guidelines checklist for surgical videos. We have presented a method for LSG video reporting, of which a similar approach can be applied to report other surgical videos. The overall inter-rater agreement, along with the agreement between surgeons and trainees at various levels of expertise, indicates that the checklist functions effectively across different skill sets. This suggests that the checklist is accessible and valuable not only for experienced surgeons but also for those with less expertise, including trainees. Consequently, this tool could be useful in helping both surgeons and trainees monitor their performance and identify any changes or improvements over time. The utility of this checklist across diverse levels of surgical expertise highlights the potential of video-based quality assessment as a broad-based tool for enhancing surgical proficiency and outcomes. Therefore, a new initiative was initiated to move the medical field forward in televisual education. The SPRINT (Standards for Presenting and Reporting clinical InterveNtions Televisually) guideline is currently under development as a universal checklist for all clinical interventional videos, registered with the EQUATOR (Enhancing the QUAlity and Transparency Of health Research) network (www.sprintguidelines.com). The aim of SPRINT is to improve the reliability and quality of published video research by promoting transparency and quality of surgical videos in the literature.

The applications of surgical videos are broad and variable, having a strong impact on clinical practice and patient outcome. Intraoperative video-based technical skills assessment, analysed in a systematic review by Bavardi et al. [22], supported the association between superior surgical techniques and lower postoperative morbidity. The authors proposed video analysis as an approach to surgical quality improvement. There is a significant opportunity to improve e-learning strategies through robust studies with clear reporting outcomes and standardized metrics. Some resources such as WebSurg IRCAD’s online University has proven to be a high-quality, peer reviewed e-learning resource which adapts to LAP-VEGaS guidelines [23, 24] providing content that meets Health on the Net Foundation (HONCode) ethical requirements for quality, confidentiality, neutrality, transparency, community and visibility, guaranteeing reliable and expert health information [25]. These peer reviewed and readily available educational videos are becoming an important part of surgical education. Beyond educational importance of videos, there are other essential benefits from recording operative procedures. Videos can potentially identify errors or complications, and therefore aid in the improvement of patient safety by learning from those experiences. Moreover, videos can possibly be used in the future as documents or evidence in medicolegal cases.

One of the strengths of this study is the objectivity of the designed evidenced-based checklist to aid comparing uploaded LSG videos. However, there are important limitations that must be addressed. This study included a relatively low number of videos used in the analysis, especially from peer-reviewed literature, and a lack of expert consensus agreement of the developed reporting guidelines video checklist. It must also be taken into account that videos on commercial platforms do not undergo a strict peer-review process prior to upload onto the website.

## Conclusions

Surgical videos of LSG are increasingly becoming more important and accessible on the internet, however there is no reliable quality assessment tool available. It is possible to develop an evidence-based checklist that can assess the quality of these surgical videos based on previous research and society guidelines. The checklist presented here delivers consistent results across users of varying expertise levels, demonstrating its usability. Academic videos were overall more detailed and more commonly narrated suggesting higher quality and educational value. Most commercial videos lack important elements such as description of technique and narrations of the procedure. Furthermore, there is a lack of peer review to act as quality control on these platforms. These findings demonstrate that there is a need for a reliable quality assessment tool beyond LSG in order to promote transparent and accurate reporting of surgical videos. Future studies should aim to achieve expert consensus, through the Delphi technique, on the development of optimal video reporting guidelines in surgery.

### Supplementary Information

ESM 1 (PDF 236 kb)

## Data Availability

The authors confirm that the data supporting the findings of this study are available within the article and its supplementary materials.

## Abbreviations

BMSS: Bariatric Metabolic Surgery Standardization
GOJ: Gastroesophageal junction
LSG: Laparoscopic Sleeve Gastrectomy
PRISMA: Preferred Reporting Items for Systematic Reviews and Meta-Analyses guidelines
