# Supplementary material for: Development and Evaluation of a Quality Assessment Tool for Laparoscopic Sleeve Gastrectomy Videos: A Review and Comparison of Academic and Online Video Resources
Source: Obes Surg. 2024 Apr 6;34(5):1909–16. doi: 10.1007/s11695-024-07199-0 (PMC11031436; doi:10.1007/s11695-024-07199-0)
Supplement: Supplementary file 1 — (PDF 236 kb) [file 11695_2024_7199_MOESM1_ESM.pdf]

Supplementary File 1:

	Assessor 1 (Academic)		Assessor 1 (YouTube)		Assessor 2 (Academic)		Assessor 2 (YouTube)	
Score	**Yes**	**No**	**Yes**	**No**	**Yes**	**No**	**Yes**	**No**
Distance from pylorus	11	15	27	40	13	13	29	38
Distance from GOJ	20	6	48	19	21	5	45	22
Use of Orogastric tube	24	2	60	7	24	2	60	7
Orogastric tube size (when used)	22	2	7	53	22	2	7	53
Dissection of proximal stomach	16	10	58	9	14	22	60	7
Staple line reinforcement	16	10	31	36	18	8	30	37
Leak test	8	18	16	51	9	17	17	50
Volume of Sleeve	0	26	0	67	0	26	0	67
Stapler information (size & type)	18	8	30	37	17	9	28	39
Aim of video uploaded	26	0	67	0	26	0	67	0
Complications/30-day outcome	14	12	0	67	14	12	0	67

	Assessor 3 (Academic)		Assessor 3 (YouTube)		Assessor 4 (Academic)		Assessor 4 (YouTube)	
Score	**Yes**	**No**	**Yes**	**No**	**Yes**	**No**	**Yes**	**No**
Distance from pylorus	10	12	22	41	11	11	33	30
Distance from GOJ	16	6	27	36	15	7	27	36
Use of Orogastric tube	15	7	56	7	15	7	56	7
Orogastric tube size (when used)	13	9	8	55	13	9	12	51
Dissection of proximal stomach	18	4	59	4	16	6	57	6
Staple line reinforcement	16	6	28	35	16	6	28	35
Leak test	8	14	12	51	8	14	13	50
Volume of Sleeve	0	22	3	60	0	22	6	57
Stapler information (size & type)	15	7	20	43	14	8	21	42
Aim of video uploaded	21	1	62	1	21	1	56	7
Complications/30-day outcome	13	9	2	61	13	9	3	60

	Assessor 5 (Academic)		Assessor 5 (YouTube)		Assessor 6 (Academic)		Assessor 6 (YouTube)	
Score	**Yes**	**No**	**Yes**	**No**	**Yes**	**No**	**Yes**	**No**
Distance from pylorus	8	14	24	39	12	10	23	40
Distance from GOJ	14	8	30	63	17	5	32	31
Use of Orogastric tube	15	7	56	7	15	7	56	7
Orogastric tube size (when used)	13	9	9	54	10	12	9	54
Dissection of proximal stomach	18	4	59	4	18	4	60	3
Staple line reinforcement	16	6	28	35	16	6	28	35
Leak test	9	13	9	54	8	14	13	50
Volume of Sleeve	0	22	8	55	1	21	3	60
Stapler information (size & type)	15	7	23	40	15	7	25	38
Aim of video uploaded	21	1	61	2	21	1	58	5
Complications/30-day outcome	13	9	2	61	13	9	2	61
